# HMGA1 regulates the mitochondrial apoptosis pathway in sepsis-induced cardiomyopathy

**DOI:** 10.1007/s12013-024-01236-6

**Published:** 2024-03-02

**Authors:** Jing Xu, Xinwei Li, Qianqian Lu, Xiaohua Li, Hongying Shan

**Affiliations:** 1 The First Affiliated Hospital of Shihezi University, Xinjiang, China; 2 Changji Branch, First Affiliated Hospital of Xinjiang Medical University, Xinjiang, China; 3 Medical School of Shihezi University, Xinjiang, China; 4 Department of Obstetrics and Gynecology, Peking University Third Hospital, Beijing, China

**Keywords:** HMGA1, sepsis-induced cardiomyopathy, lipopolysaccharide, mitochondria, apoptosis

## Abstract

High mobility group protein AT-hook 1 (HMGA1), an architectural transcription factor, has previously been reportedto play an essential role in architectural remodeling processes. However, its effects on cardiovascular diseases, particularly sepsis-induced cardiomyopathy, have remained unclear. The study aimed to investigate the role of HMGA1 in lipopolysaccharide-induced cardiomyopathy. Mice subjected to lipopolysaccharide for 12 h resulted in cardiac dysfunction. We used an adeno-associated virus 9 delivery system to achieve cardiac-specific expression of the HMGA1 gene in the mice. H9c2 cardiomyocytes were infected with Ad-HMGA1 to overexpress HMGA1 or transfected with si-HMGA1 to knock down HMGA1. Echocardiography was applied to measure cardiac function. RT-PCR was used to detect the transcriptional level of inflammatory cytokines. CD45 and CD68 immunohistochemical staining were used to detect inflammatory cell infiltration and TUNEL staining to evaluate the cardiomyocyte apoptosis, MitoSox was used to detect mitochondrial reactive oxygen species, JC-1 was used todetect Mitochondrial membrane potential. Our findings revealed that the overexpression of HMGA1 exacerbated myocardial inflammation and apoptosis in response to lipopolysaccharide treatment. Additionally, we also observed that H9c2 cardiomyocytes with HMGA1 overexpression exhibited enhanced inflammation and apoptosis upon stimulation with lipopolysaccharide for 12 h. Conversely, HMGA1 knockdown in H9c2 cardiomyocytes attenuated lipopolysaccharide-induced cardiomyocyte inflammation and apoptosis. Further investigations into the molecular mechanisms underlying these effects showed that HMGA1 promoted lipopolysaccharide-induced mitochondrial-dependent cardiomyocyte apoptosis. The study reveals that HMGA1 worsens myocardial inflammation and apoptosis in response to lipopolysaccharide treatment. Mechanically, HMGA1 exerts its effects by regulating the mitochondria-dependent apoptotic pathway.

## Introduction

Sepsis-induced cardiomyopathy (SIC) is a severe complication of sepsis, characterized by significant cardiac dysfunction in septic patients. It is manifested by a reduction in left ventricular ejection fraction, decreased shortening fraction, intractable heart failure, and diastolic and systolic dysfunction, which are major causes of high mortality rates in ICU patients [1]. The pathogenesis of SIC is complex and involves dysregulation of inflammatory factors, mitochondrial dysfunction, oxidative stress, and cell apoptosis [2, 3].

High mobility group protein A1 (HMGA1) binds to AT-rich DNA regions [4] and regulates various fundamental cellular processes at the molecular and cellular levels, including cell cycle regulation [5], embryonic development [6], tumor transformation [7], cell proliferation and differentiation [8]. Recent studies have demonstrated the significant role of HMGA1 in diverse cardiovascular diseases. For example, inhibition of HMGA1/NF-κB signaling attenuated myocardial injury induced by coronary microembolization (CME) and improved cardiac function, highlighting a potential therapeutic target for the prevention and treatment of CME-induced myocardial injury [9]. HMGA1 can also exacerbate isoproterenol and angiotensin II-induced myocardial fibrosis through the modulation of FOXO1 transcription [10]. Additionally, HMGA1 has been associated with isoproterenol-induced cardiac hypertrophy [11]. Hopper et al. reported enhanced expression of HMGA1 in pulmonary arterial endothelial cells of patients with idiopathic pulmonary arterial hypertension (PAH), and the upregulation of HMGA1 promoted endothelial-mesenchymal transition, accelerating the progression of PAH [12]. Moreover, HMGA1 can inhibit autophagy, ultimately leading to cardiac dysfunction in diabetic mice, as well as inducing inflammation and apoptosis in high-glucose-induced cardiomyocytes [13].

However, the role of HMGA1 in lipopolysaccharide (LPS)-induced cardiomyocyte apoptosis remains unexplored. This study aims to investigate the role and mechanism of HMGA1 in LPS-induced cardiomyocyte apoptosis.

## Methods

### Materials

Lipopolysaccharide (LPS) from Escherichia coli O55:B5 was purchased from Sigma (USA). Adeno-associated virus 9 carrying wild-type HMGA1 gene (AAV9-HMGA1) and green fluorescent protein (AAV9-GFP) was constructed by Vigene Bioscience (Shandong, China). Adenovirus carrying wild-type HMGA1 gene (Ad-HMGA1) and green fluorescent protein (Ad-GFP) were generated from Vigene Bioscience (Shandong, China). Negative siRNA (si-NC) and HMGA1 siRNA (si-HMGA1) were obtained from Ribo Life Science (Suzhou, China). The anti-HMGA1 antibody (ab129153) is purchased from ABCAM company(UK). TRIzol reagent was purchased from Invitrogen (USA). Reverse transcription kit and SYBR Green I Master were purchased from Roche (Switzerland). CD45 antibody and CD68 antibody were purchased from Cell Signaling Technology (USA). Lipofectamine RNAiMAX transfection reagent was obtained from Thermo Fisher(USA). ApopTag® Plus In situ Apoptosis Fluorescein Detection Kit (S7111) was bought from Millipore Corporation (USA). Mitochondrial membrane potential detection kit (JC-1) and was purchased from Shanghai Biyuntian; MitoSOX Red Mitochondrial Superoxide Indicator was purchased from Invitrogen (USA). CellTiter-Glo®Luminescent Cell Viability Assay kit and Caspase-Glo® 3/7 Assay kit was obtained from Promega (USA).

### Mice

Eight to ten-week-old male C57BL/6 J mice, weighing between 23.5–27.5 g, were purchased from the Institute of Laboratory Animal Sciences, Chinese Academy of Medical Sciences, and housed in a specific-pathogen-free (SPF) animal facility. The mice were randomly divided into four groups: saline+AAV9-GFP group, saline+AAV9-HMGA1 group, LPS + AAV9-GFP group, and LPS + AAV9-HMGA1 group, with 15 mice in each group. The saline+AAV9-HMGA1 group and LPS + AAV9-HMGA1 group were injected intramyocardially with 1×10^11 viral genome particles per mouse of AAV9-HMGA1. Saline+AAV9-GFP group and LPS + AAV9-GFP group were injected intramyocardially with AAV9-GFP. After one week of myocardial viral injection, the mice received intraperitoneal injection of 10 mg/kg LPS in saline solution for 12 h. The mice were euthanized by cervical dislocation, followed by rapid thoracotomy. The intact hearts were quickly removed, fixed in 10% formaldehyde solution, dehydrated, embedded in paraffin, and then sectioned into 5 μm thick tissue slices.

### Cell culture and treatment

The H9c2 cardiomyocyte cell line was purchased from the Cell Bank of the Chinese Academy of Sciences. The cells were cultured in DMEM medium containing 10% FBS and incubated at 37 °C in a humidified incubator with 5% CO_2_. When the cell density reached approximately 80%, the cells were digested with 0.25% trypsin for passage. H9c2 cardiomyocytes were infected with Ad-HMGA1 (MOI = 30) for 8 h to overexpress HMGA1 or transfected with si-HMGA1 for 12 h to knock down HMGA1. After that, H9c2 cardiomyocytes were stimulated with 1 μg/mL LPS for 12 h to induce inflammation.

### Echocardiography

Prior to the experiment, the mouse’s chest hair should be shaved and it should be positioned in a supine manner on a detection table for ultrasound imaging. A suitable quantity of ultrasound coupling agent should be applied to the front area of the chest. The ultrasound probe should be gently placed on the coupling agent, positioned tangentially to the long axis of the mouse’s heart. Then, the position and direction of the probe should be adjusted until the short-axis section of the heart’s papillary muscle plane is clearly displayed. M-mode ultrasound should be employed to evaluate the left ventricular ejection fraction. Once the mouse’s breathing becomes stable, the imaging of the cardiac cycle and ventricular chamber movement should be recorded.

### Western-blot

The mouse heart tissue or H9c2 cardiomyocytes from each experimental group were lysed in RIPA buffer. The resulting supernatant was collected following sonication and high-speed centrifugation. Protein concentrations were determined using the BCA Protein Assay Kit. Subsequently, the protein samples were separated on a 10% SDS-PAGE gel and transferred onto PVDF membranes. The membranes were then incubated overnight at 4 °C with specific primary antibodies, followed by incubation with secondary antibodies at 37 °C for 1 h. Finally, the protein bands were visualized using the ECL chemiluminescent system.

### Total RNA isolation and quantitative real-time PCR

We extracted total RNA from left ventricle tissues and cultured cardiomyocytes using TRIzol® reagent from Invitrogen, following the manufacturer’s instructions. This RNA was then reverse-transcribed into cDNA using the Transcriptor First Strand cDNA Synthesis Kit by Roche. Quantitative real-time PCR amplification was performed using the SYBR Green PCR Master Mix provided by Applied Biosystems. Each PCR reaction was conducted in triplicate, and the results are presented as the mean of relative gene expression normalized to GAPDH gene expression. Primer sequences employed in this research in Table 1.

**Table 1:** The sequences of the primers for each gene

Gene(species)	Forward primer	Reverse primer
GAPDH(mouse)	TGTGAACGGATTTGGCCGTA	GATGGTGATGGGTTTCCCGT
HMGA1(mouse)	GGTCGGGAGTCAGAAAGAGC	ATTCTTGCTTCCCTTTGGTCG
IL-1β (mouse)	GCAACTGTTCCTGAACTCAACT	ATCTTTTGGGGTCCGTCAACT
IL-6 (mouse)	AAAGCAAACTGAGGGCTCTGCTCG	TTCGGTACCGGAAGCTGTTGCA
TNF-α (mouse)	AAAGCAAACTGAGGGCTCTGCTCG	TTCGGTACCGGAAGCTGTTGCA
GAPDH (rat)	TCCTGCACCACCAACTGCTTAG	AGTGGCAGTGATGGCATGGACT
HMGA1(rat)	GGATGGGACTGAGAAGCGAG	TTGTTCTTGCTTCCCTTCGG
IL-1β (rat)	CCTATGTCTTGCCCGTGGAG	CACACACTAGCAGGTCGTCA
IL-6 (rat)	CACTTCACAAGTCGGAGGCT	AGCACACTAGGTTTGCCGAG
TNF-α (rat)	GCGGGCGGCGGTAAAATG	AGGTCCACGTCTTTGCATGT

### Immunochemistry staining

After dewaxing the tissue sections with xylene and graded alcohols, antigen retrieval was performed using the high-pressure method with citric acid. After blocking with 3% hydrogen peroxide, the sections were incubated overnight at 4 °C with anti-CD45 antibody or anti-CD68 antibody. The next day, the sections were incubated with anti-rabbit EnVisionTM + /HRP reagent at room temperature for 20 min, followed by the addition of DAB working solution under an optical microscope. Finally, the nuclei were stained with hematoxylin, and the sections were dehydrated with graded alcohols and cleared with xylene before mounting.

### TUNEL staining

Apoptosis detected by TdT-mediated dUTP nick end labeling (TUNEL) methods. TUNEL staining in cardiac sections was performed using a commercial kit and operated following the manufacturer’s instructions. Briefly, the cardiac tissue sections are dewaxed in xylene and dehydrated in a gradient of ethanol. Then, proteinase K without DNase (20 μg/ml) is added to the cardiac samples, and they are incubated at room temperature for 30 min. The sections are then rinsed with PBS three times, incubated for 20 min at room temperature in a 3% hydrogen peroxide solution (3% H2O2 in PBS), and subsequently rinsed three times with PBS. Post addition of 50 μl of biotin-labeled solution, the samples are rinsed with PBS, followed by the addition of 0.2 ml of labeling reaction termination solution. After that, the samples are incubated at room temperature for 10 min before being rinsed three times with PBS. Subsequently, 50 μl of Streptavidin-HRP working solution is added, and the samples are incubated at room temperature for 30 min, followed by three rinses with PBS. Finally, 0.3 ml of DAB staining solution is added, and the cell nuclei are stained with hematoxylin staining solution.

For the cell, TUNEL staining was performed to observe the apoptosis index following the standard protocol using the ApopTag® Plus Fluorescein in situ Apoptosis Detection Kit. the cells were treated with 1% paraformaldehyde for 10 min at room temperature. After that, a mixture of ethanol and acetic acid was applied for 5 min. Then, the cells were supplemented with the equilibration buffer at room temperature, and then treated with the TdT Enzyme working solution and the Stop/WashBuffer working solution. After incubating the cells with the Anti-Digoxigenin Fluorescein working solution for 30 min in the dark, the samples were sealed using SlowFade Gold Antifade Reagent (with DAPI) to preserve the fluorescence.

### CK and LDH

After 12 h of lipopolysaccharide injection, mouse orbital venous blood was taken, left to stand for 30 min, and then centrifuged at 3000 rpm for 10 min at room temperature to obtain the supernatant. According to the manufacturer’s instructions, lactate dehydrogenase (LDH) and creatine kinase isoenzyme (CK-MB) levels were detected. In short, 2 μl of serum was diluted 20 times to 40 μl with ddH2O and added to a 96-well reaction plate. Immediately add 200 μl of LDH or CK-MB detection working solution and place it in a microplate reader for immediate detection. Measure the UV absorbance at a wavelength of 340 nm, read the OD value every minute for a total of 7 times, calculate the slope of OD values for each well in 7 consecutive readings, and calculate the relative change and absolute quantity of LDH or CK-MB. Absolute change value (U/L) = slope × 3376 × 20.

### RNA interference

The siRNA was transfected in H9c2 cardiomyocytes using Lipofectamine RNAiMAX transfection reagent. After mixing siRNA and transfection reagent separately with opt-MEM, let them stand for 5 min. After mixing the aforementioned two reagents together and gently stirring, let the mixture stand for 10 min. Then, add the aforementioned mixture to the cell culture medium.

### JC-1 assay

The JC-1 mitochondrial membrane potential detection kit was applied to detect mitochondrial membrane potential changes. When the mitochondrial membrane potential is at a high level, JC-1 aggregates inside the mitochondria and turns into red fluorescence. When the mitochondrial membrane potential is at a low level, JC-1 cannot accumulate inside the mitochondria and remains in the cytoplasm as green fluorescent monomers. To perform the staining, the culture medium in the 6-well plate was removed, and a mixture of JC-1 staining working solution and cell culture medium in a 1:1 ratio was added. The plate was then incubated at 37 °C in a dark room for 20 min. After washing twice with JC-1 staining buffer, cell culture medium was added, and the cells were observed and photographed under a fluorescence microscope.

### MitoSox staining

The MitoSox assay was used to detect mitochondrial reactive oxygen species (ROS) production. The MitoSox dye is capable of permeating live cells and selectively targeting mitochondria. It undergoes specific oxidation by mitochondrial ROS, generating a red fluorescent product when it binds to nucleic acid. The cells were washed with PBS and stained with 5 μM MitoSOX Red at 37 °C for 30 min, protected from light. After washing with PBS, the cells were observed and photographed under a fluorescence microscope.

### ATP and Caspase 3/7 activity

ATP is detected using the CellTiter-Glo® Luminescent Cell Viability Assay, while Caspase 3/7 activity is measured using the Caspase-Glo® 3/7 Assay. Cells are seeded in a 96-well plate and treated according to the experimental design, with the removal of the culture medium prior to testing. The assay reagents are mixed with PBS buffer in appropriate volumes, and 120 μl is added to each well. The plate is placed on a shaker and incubated for 30 min, avoiding light exposure. Then, 100 μl of the reaction mixture is transferred to a solid white, opaque 96-well plate to measure the intensity of the luminescent signal produced by the luciferase reaction. The relative value of caspase 3/7 activity is calculated.

### Statistical analysis

The experimental data are presented as mean ± standard deviation (SD). To analyze the results of two groups, an unpaired t-test was conducted, assuming normal distribution for both groups. Differences were considered significant when the p-value was less than 0.05. For comparisons among three or more groups, a one-way ANOVA was performed, and a subsequent multiple comparison test was conducted.

## Results

### Overexpression of HMGA1 exacerbates inflammation in LPS-induced heart tissue

**Fig. 1:**
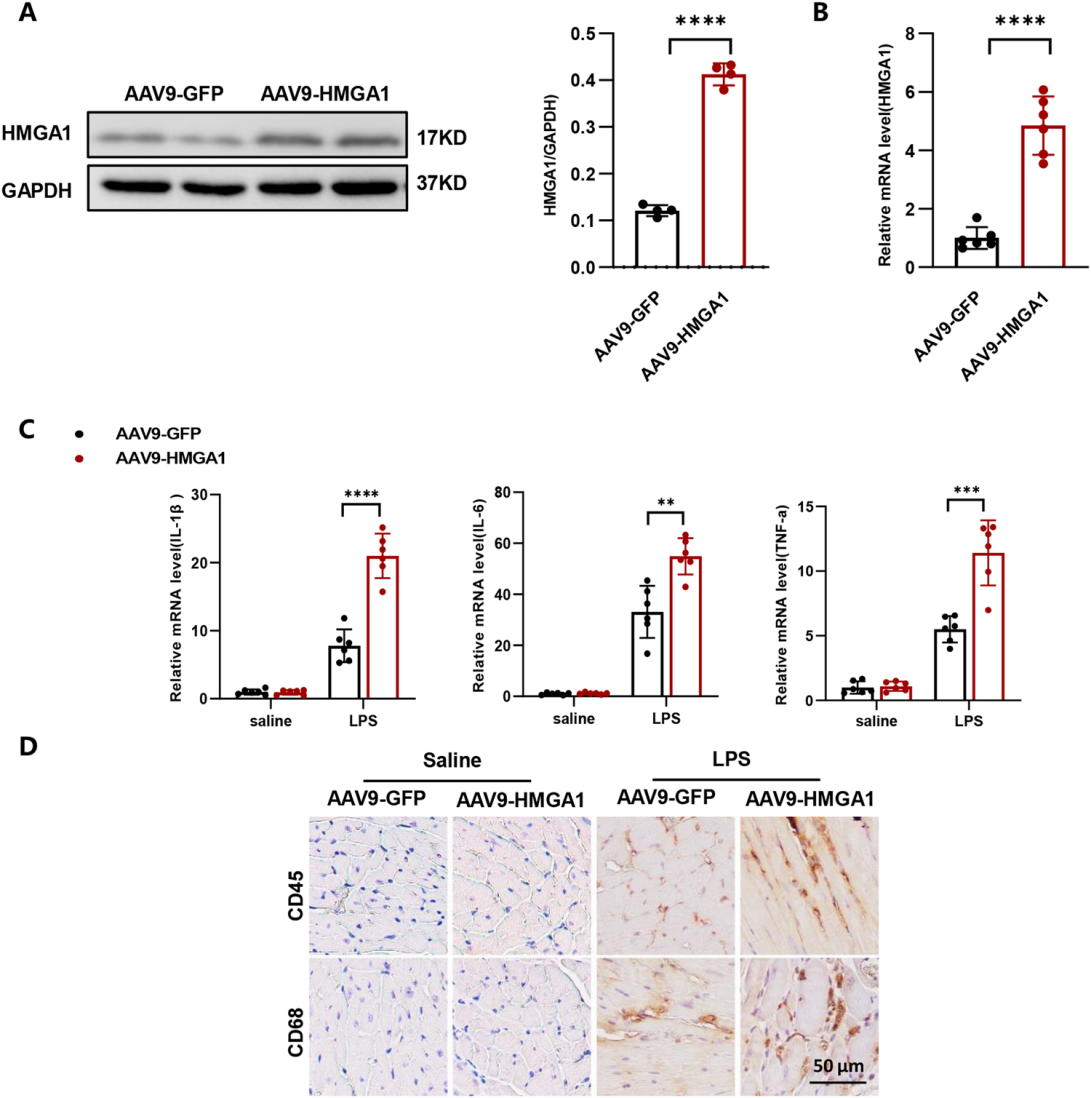
Overexpression of HMGA1 exacerbates inflammation in LPS-induced heart tissue. **A** Western blot image and quantification of HMGA1 in heart tissue after adeno-associated virus injection (*n* = 4). **B** The relative mRNA expression level of HMGA1 in the heart tissue following adeno-associated virus infection (*n* = 6). **C** The relative mRNA levels of IL-1β, IL-6, TNF-α among groups (*n* = 6). **D** Representative images of immunochemistry staining from hearts in the indicated groups (*n* = 6, scale bar, 50 μm). Values represent the mean ± SD. *p < 0.05; **p < 0.01, ***p < 0.001; ****p < 0.0001

To explore the involvement of HMGA1 in LPS-induced myocardial inflammation, we initially administered HMGA1 adenovirus into the myocardium of mice to induce targeted overexpression of HMGA1 in cardiac tissues (shown in Fig. 1A, B). Our findings indicate that a 12-hour LPS treatment exacerbates inflammation in the cardiac tissues, as evidenced by the upregulated transcription levels of inflammatory cytokines, such as IL-1β, IL-6, TNF-α (shown in Fig. 1C). And the overexpression of HMGA1 in cardiac cells results in an increase in the levels of inflammatory cytokines (shown in Fig. 1C). At the same time, immunohistochemical staining results also showed a significant increase in CD45-positive leukocytes and CD68-positive macrophages in the heart under LPS stimulation (shown in Fig. 1D). The above results indicate that cardiac-specific overexpression of HMGA1 exacerbated inflammatory cell infiltration in the heart tissue.

### Overexpression of HMGA1 exacerbates apoptosis in LPS-stimulated heart tissue

**Fig. 2:**
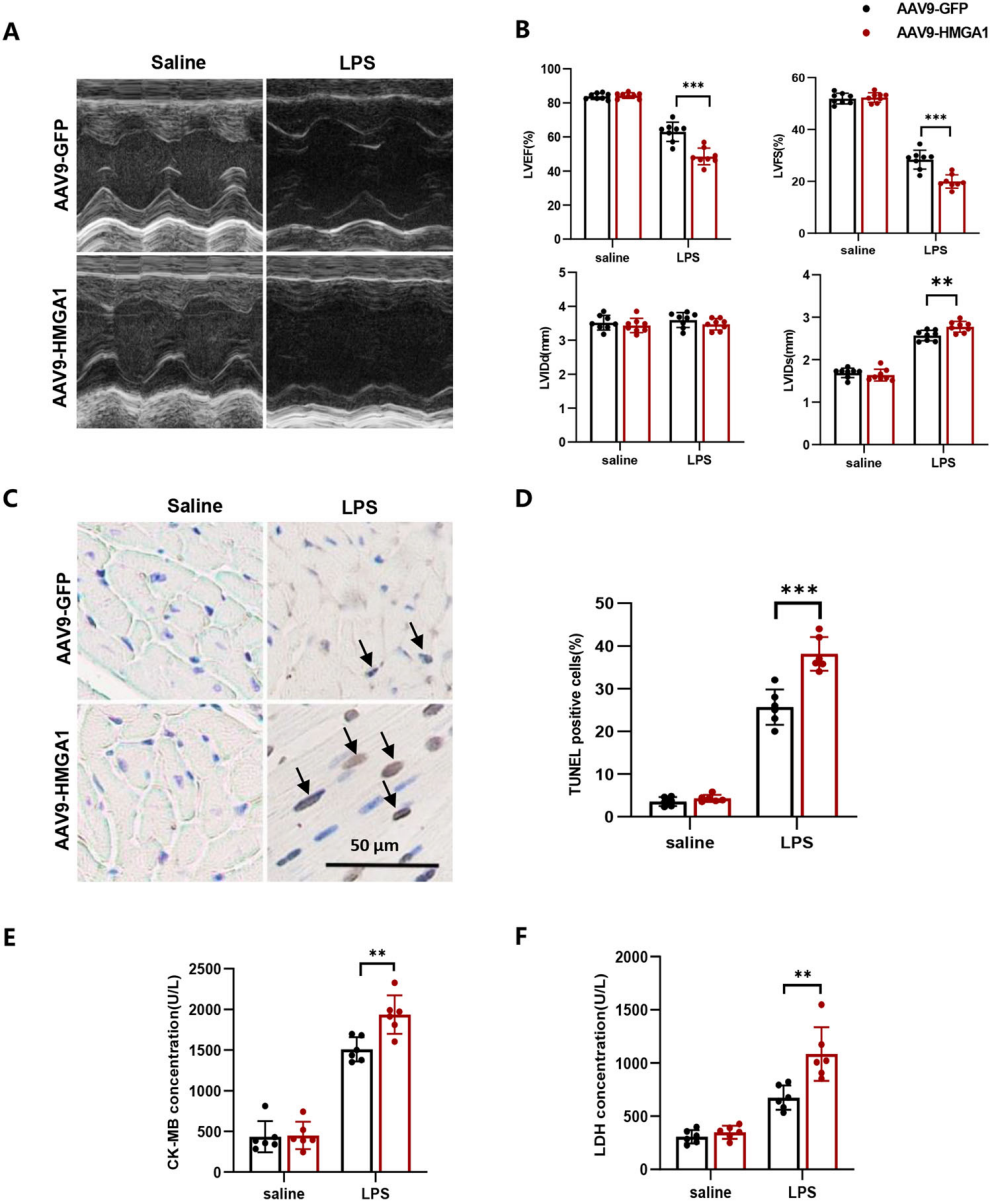
Overexpression of HMGA1 exacerbates apoptosis in LPS-stimulated heart tissue. **A** Cardiac function of mice injected with AAV9-HMGA1or AAV9-GFP was analyzed by transthoracic echocardiography (*n* = 8). **B** Statistical results of left ventricular ejection fraction (LVEF), left ventricular fractional shortening (LVFS), left ventricle internal diameters at diastole (LVIDd), systole (LVIDs) in the indicated groups. **C** Representative images of TUNEL staining in heart samples (*n* = 6), and black arrows indicate TUNEL-positive nuclei. **D** The quantitative data about TUNEL-positive nuclei in heart samples (*n* = 6). **E** Circulating levels of CK-MB in mice with AAV9-HMGA1or AAV9-GFP injection (*n* = 6). **F** Circulating levels of LDH in mice with AAV9-HMGA1or AAV9-GFP injection (*n* = 6). Values represent the mean ± SD. *p < 0.05; **p < 0.01, ***p < 0.001; ****p < 0.0001

Our study found that LPS stimulation for 12 h led to deterioration of mouse cardiac function, as reflected by decreased left ventricular ejection fraction and short-axis shortening rate, and increased end-systolic and end-diastolic left ventricular diameter (shown in Fig. 2A, B). Cardiac-specific overexpression of HMGA1 further aggravated cardiac dysfunction (shown in Fig. 2A, B). In addition, TUNEL staining showed that the apoptosis rate of cardiomyocytes under LPS treatment was significantly higher than that in the control group, and HMGA1 further exacerbated the degree of cardiomyocytes apoptosis (shown in Fig. 1C, D). In addition, HMGA1 aggravated LPS-induced myocardial injury, manifested as increased levels of serum CK-MB and LDH (shown in Fig. 1E, F).

### HMGA1 exacerbates the inflammatory response in H9c2 cardiomyocytes treated with LPS

**Fig. 3:**
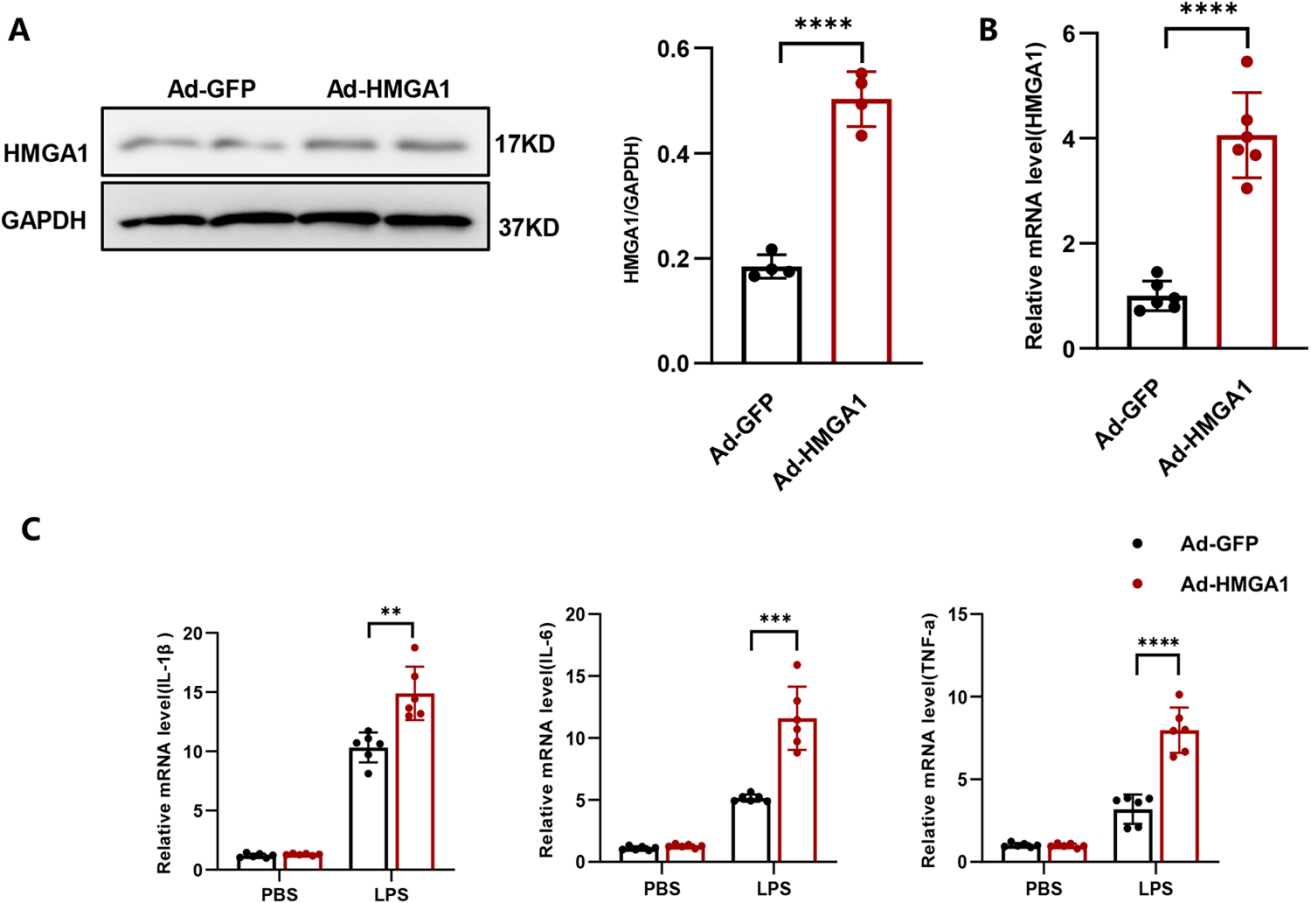
HMGA1 exacerbates the inflammatory response in H9c2 cardiomyocytes treated with LPS. **A** Western blot image and quantification of HMGA1 in H9c2 cardiomyocytes transfected with Ad-HMGA1 (*n* = 4). **B** The relative mRNA expression level of HMGA1 in H9c2 cardiomyocytes following adenovirus transfection (*n* = 6). **C** Relative mRNA levels of IL-1β, IL-6, TNF-α in LPS-treated H9c2 cardiomyocytes after infection with Ad-HMGA1 were determined by real-time PCR assays (*n* = 6). Values represent the mean ± SD. *p < 0.05; **p < 0.01, ***p < 0.001; ****p < 0.0001

In order to gain further insight into the functional role of HMGA1, adenovirus was used to induce overexpression of HMGA1 in H9c2 cardiomyocytes (shown in Fig. 3A, B). Subsequently, the H9c2 cardiomyocytes were stimulated with LPS for a duration of 12 h. This resulted in a significant increase in the inflammatory response of the H9c2 cardiomyocytes, as indicated by the elevated transcription levels of IL-1β, IL-6, and TNF-α (shown in Fig. 3C) Remarkably, the overexpression of HMGA1 further aggravated the inflammatory response induced by LPS (shown in Fig. 3C).

### HMGA1 exacerbates mitochondrial-mediated cell apoptosis in H9c2 cardiomyocytes treated with LPS

**Fig. 4:**
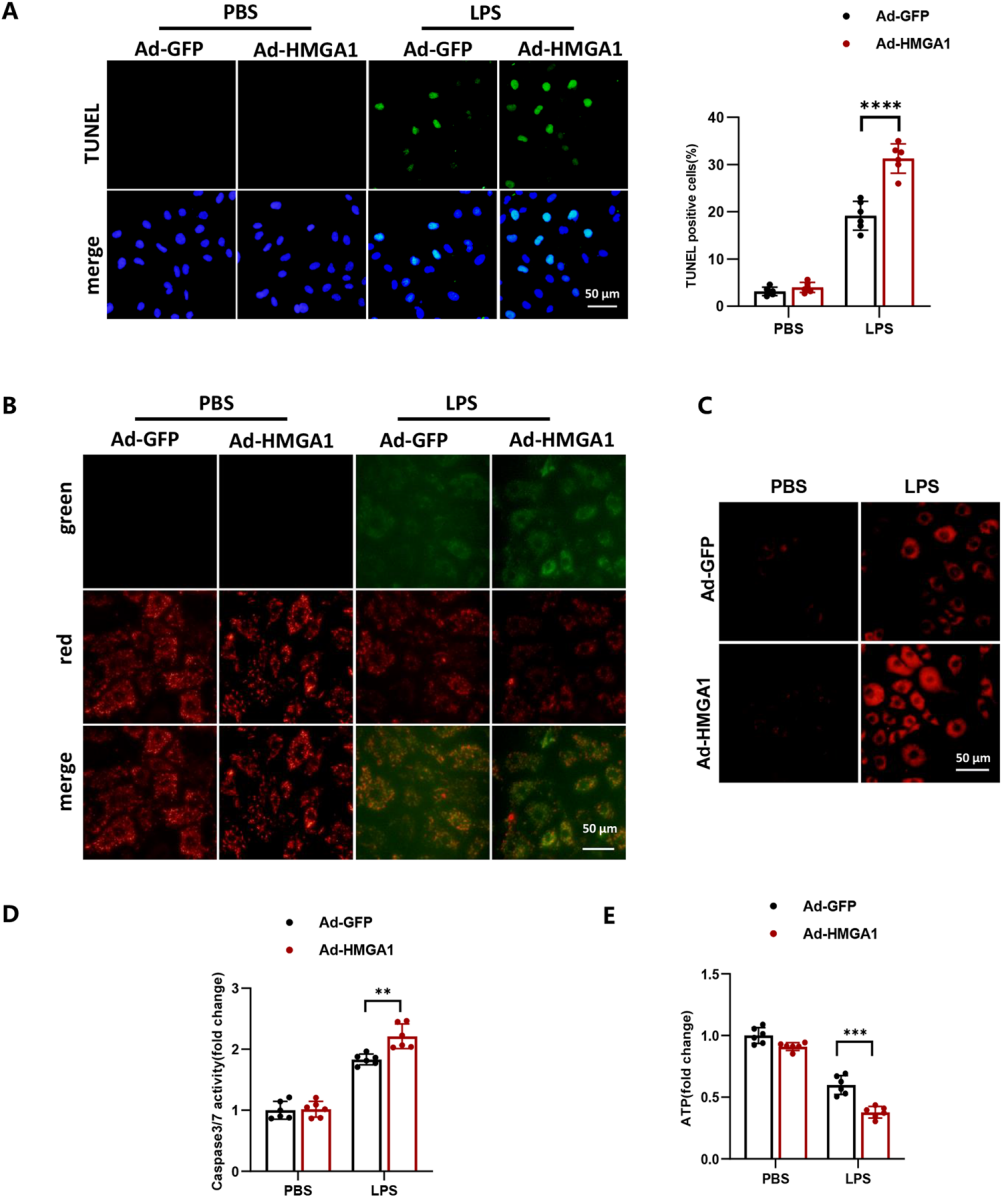
HMGA1 exacerbates mitochondrial-mediated cell apoptosis in H9c2 cardiomyocytes treated with LPS. **A** Representative TUNEL images of H9c2 cardiomyocytes infected with Ad-HMGA1or Ad-GFP and treated with LPS (1 μmol/l) or PBS for 12 h (*n* = 6; blue, nucleus; green, apoptosis-positive cells; scale bar, 50 μm). Right: quantitative results. **B** Change of mitochondrial membrane potential in Ad-HMGA1-infected H9c2 cardiomyocytes with LPS treatment was detected by a JC-1 fluorescent probe, with red fluorescence indicating high mitochondrial membrane potential and green indicating low mitochondrial membrane potential (*n* = 6, scale bar,50 μm). **C** Production of the mitochondrial superoxide anion (mitochondrial O2˙ − ) LPS-treated H9c2 cardiomyocytes after infection with Ad-HMGA1 was measured using MitoSOX™ Red (*n* = 6, scale bar,50 μm). **D** Relative caspase3/7 activity in Ad-HMGA1-infected H9c2 cardiomyocytes with LPS treatment (*n* = 6). **E** Relative amount of ATP in Ad-HMGA1-infected H9c2 cardiomyocytes with LPS treatment (*n* = 6). Values represent the mean ± SD. *p < 0.05; **p < 0.01, ***p < 0.001; ****p < 0.0001

TUNEL staining showed that the proportion of apoptotic cells increased under LPS stimulation, and HMGA1 further exacerbated cell apoptosis (shown in Fig. 4A). JC-1 detection results showed that the red fluorescence of cells in the LPS group was weakened and the green fluorescence was enhanced compared with the control group, and HMGA1 overexpression exacerbated this phenomenon (shown in Fig. 4B), indicating a decrease in mitochondrial membrane potential after HMGA1 overexpression. MitoSox detection results showed that the red fluorescence of cells in the LPS group was increased compared with the control group, and the red fluorescent signal of Ad-HMGA1 treated cells was further enhanced (shown in Fig. 4C), suggesting an increase in mitochondrial reactive oxygen species levels after HMGA1 overexpression. In addition, our results demonstrated that LPS stimulation leads to an increase in caspase3/7 activity and HMGA1 overexpression could cause a further increase in caspase3/7 activity (shown in Fig. 4D). In LPS-treated H9c2 cardiomyocytes, the relative amount of ATP is suppressed, and the overexpression of HMGA1 additionally restricts the production of ATP (shown in Fig. 4E).

In addition, our results demonstrated that LPS stimulation leads to an increase in caspase3/7 activity and HMGA1 overexpression could cause a further increase in caspase3/7 activity (shown in Fig. 2D). In LPS-treated H9c2 cardiomyocytes, the relative amount of ATP is suppressed, and the overexpression of HMGA1 additionally restricts the production of ATP (shown in Fig. 2E).

### The inhibition of HMGA1 alleviates inflammation in H9c2 cardiomyocytes treated with LPS

**Fig. 5:**
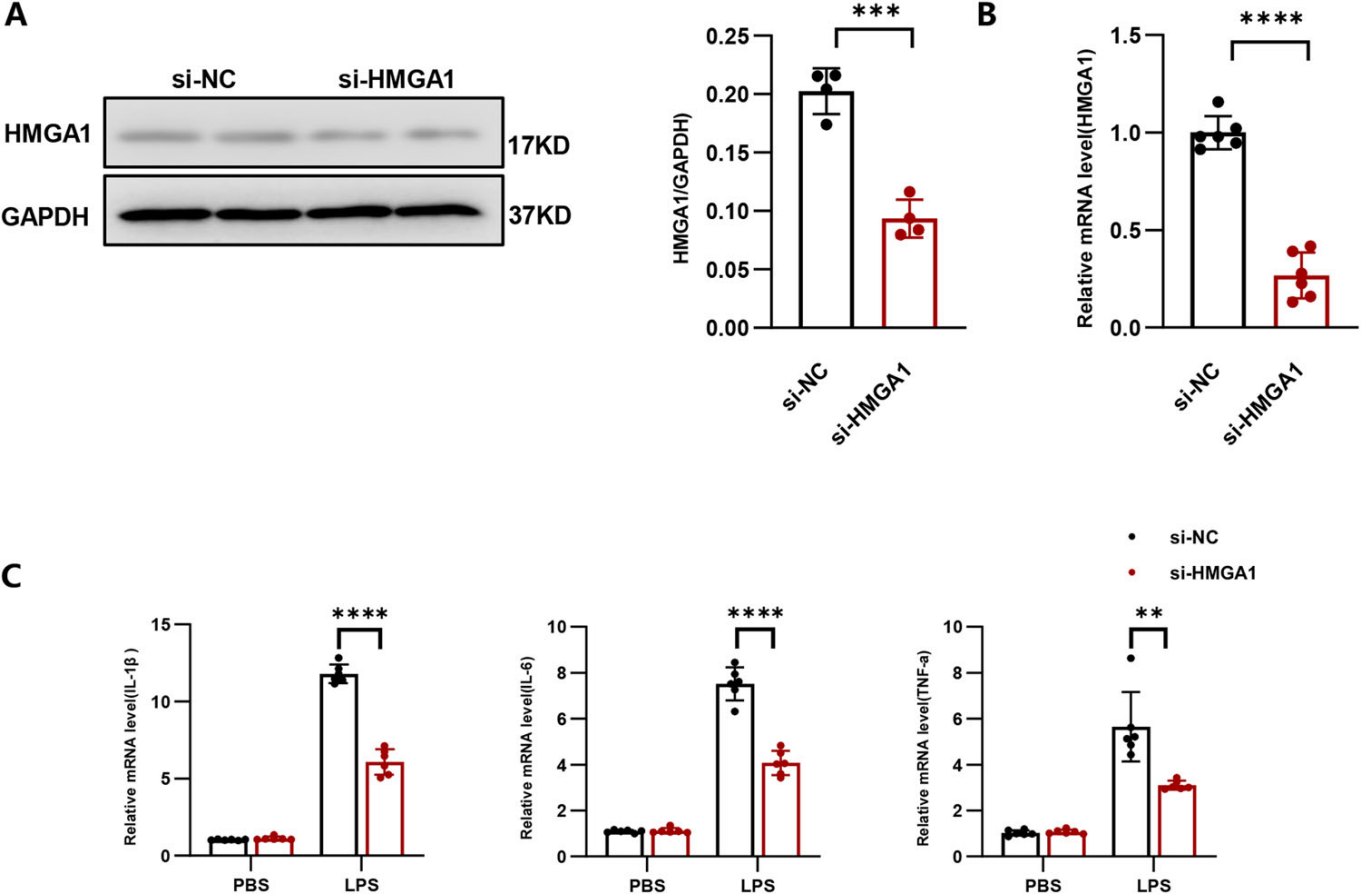
The inhibition of HMGA1 alleviates inflammation in H9c2 cardiomyocytes treated with LPS. **A** Western blot image and quantification of HMGA1 in H9c2 cardiomyocytes transfected with siRNA (*n* = 4). **B** The relative mRNA expression level of HMGA1 in H9c2 cardiomyocytes following siRNA transfection (*n* = 6). **C** Relative mRNA levels of IL-1β, IL-6, TNF-α in LPS-treated H9c2 cardiomyocytes after infection with si-HMGA1 were determined by real-time PCR assays (*n* = 6). Values represent the mean ± SD. *p < 0.05; **p < 0.01, ***p < 0.001; ****p < 0.0001

In this study, we employed siRNA to knock down HMGA1 (shown in Fig. 5A, B) in H9c2 cardiomyocytes and subsequently induced inflammation by stimulating the cells with LPS for a duration of 12 h. The experimental results demonstrated that silencing HMGA1 led to a reduction in cardiac inflammatory response to LPS (shown in Fig. 5C). These findings indicate that silencing HMGA1 may confer protection against the inflammatory processes in cardiomyocytes.

### The inhibition of HMGA1 alleviates mitochondrial-mediated cell apoptosis in H9c2 cardiomyocytes treated with LPS

**Fig. 6:**
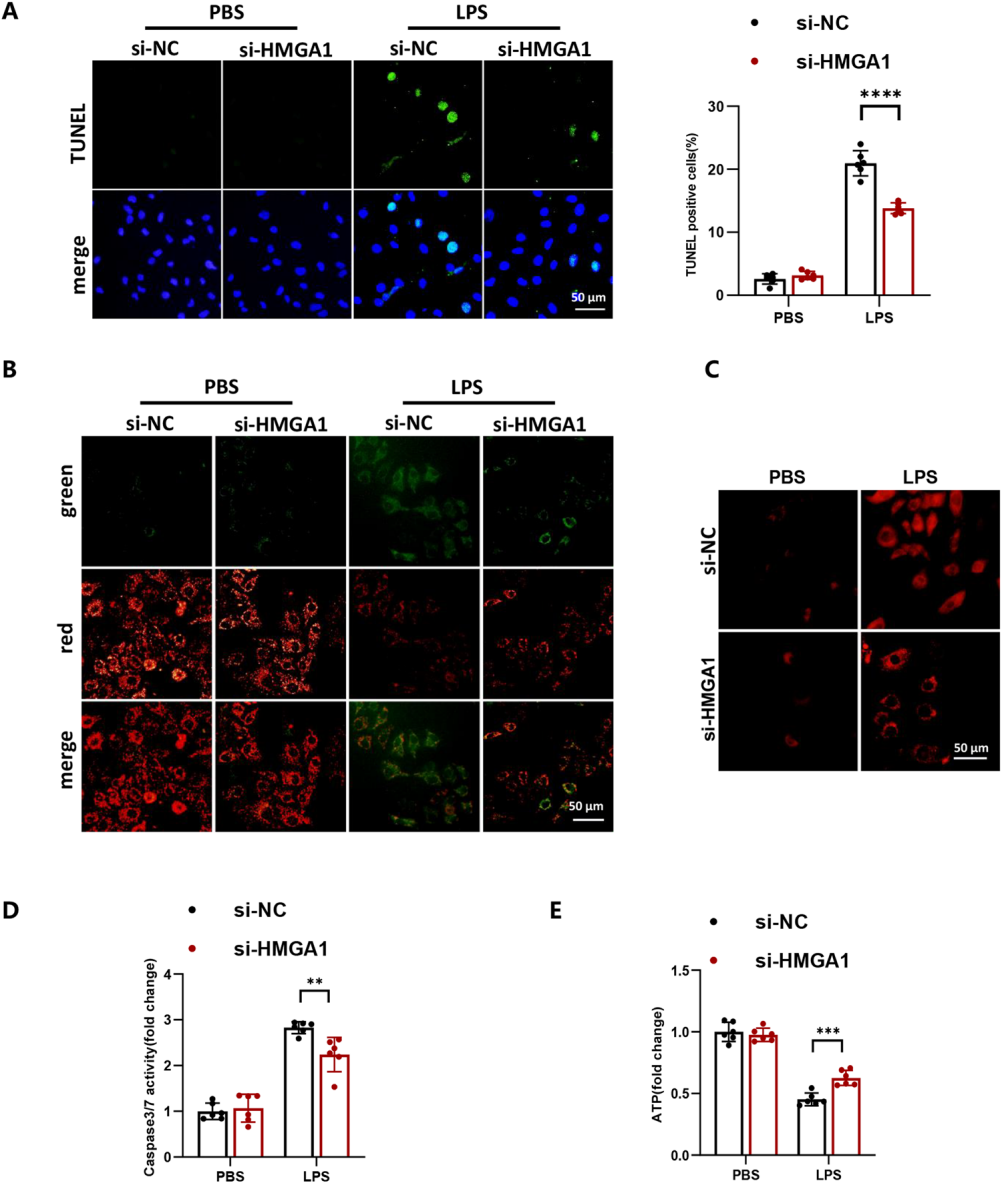
The inhibition of HMGA1 alleviates mitochondrial-mediated cell apoptosis in H9c2 cardiomyocytes treated with LPS. **A** Representative TUNEL images of H9c2 cardiomyocytes infected with si-HMGA1or si-NC and treated with LPS (1 μmol/l) or PBS for 12 h (*n* = 6; blue, nucleus; green, apoptosis-positive cells; scale bar, 50 μm). Right: quantitative results. **B** Change of mitochondrial membrane potential in si-HMGA1-infected H9c2 cardiomyocytes with LPS treatment was detected by a JC-1 fluorescent probe, with red fluorescence indicating high mitochondrial membrane potential and green indicating low mitochondrial membrane potential (*n* = 6, scale bar, 50 μm). **C** Production of the mitochondrial superoxide anion (mitochondrial O2˙ − ) LPS-treated H9c2 cardiomyocytes after infection with si-HMGA1 was measured using MitoSOX™ Red (*n* = 6, scale bar, 50 μm). **D** Relative caspase3/7 activity in si-HMGA1-infected H9c2 cardiomyocytes with LPS treatment (*n* = 6). **E** Relative amount of ATP in si-HMGA1-infected H9c2 cardiomyocytes with LPS treatment (*n* = 6). Values represent the mean ± SD. *p < 0.05; **p < 0.01, ***p < 0.001; ****p < 0.0001

TUNEL staining showed that inhibition the expression of HMGA1 alleviated cell apoptosis (shown in Fig. 6A). JC-1 detection results showed HMGA1 downregulation can lead to an increase in mitochondrial membrane potential (shown in Fig. 6B). MitoSox detection results showed that HMGA1 silencing exacerbated the production of reactive oxygen species in mitochondria (shown in Fig. 6C). Additionally, our data indicated that the increase in caspase3/7 activity stimulated by LPS treatment was inhibited by HMGA1 silence (shown in Fig. 6D). And silencing HMGA1 increased the relative amount of ATP, which is suppressed by LPS treatment (shown in Fig. 6E).

## Discussion

Previous studies have found that the expression of HMGA1 significantly increases in septic cardiac tissues and is upregulated in LPS-stimulated H9c2 cells. Cardiac-specific overexpression of HMGA1 further exacerbates cardiac dysfunction [14]. Additionally, overexpression of HMGA1 in neonatal rat cardiomyocytes (NRCMs) induced by high glucose worsened cell inflammation and apoptosis [13]. This study also observed that overexpression of HMGA1 increased the number of apoptotic-positive cardiomyocytes induced by LPS.

Cell apoptosis primarily occurs through three pathways: the extrinsic death receptor signaling pathway, intrinsic mitochondrial apoptosis pathway, and endoplasmic reticulum pathway. Among these, mitochondrial-mediated cell apoptosis is the classical pathway. Mitochondria serve as one of the main sites for the generation of reactive oxygen species, and abnormalities in mitochondrial structure and function can lead to the accumulation of reactive oxygen species. Excessive reactive oxygen species lead to lipid peroxidation, causing a decrease in the mitochondrial transmembrane potential [15, 16], resulting in the opening of the mitochondrial permeability transition pore (MPTP) and the release of cytochrome C from the mitochondrial intermembrane space into the cytoplasm [17]. Cytochrome C binds and activates apoptotic protease activating factor-1 (Apaf-1) and caspase-9 precursor in the cytoplasm to form an “apoptosome”. Caspase-9 is the initiator of apoptosis in the mitochondrial apoptosis pathway and, as a protease, can activate downstream apoptotic effector molecule caspase-3 [18], ultimately leading to irreversible cell apoptosis.

Previous studies have found that increased intracellular levels of HMGA1 affect mitochondrial function and mitochondrial DNA repair efficiency [19]. This study found that HMGA1 can increase mitochondrial reactive oxygen species levels, decrease mitochondrial membrane potential, and upregulate the expression of the mitochondrial apoptosis signaling pathway caspase-3, suggesting that HMGA1 may be involved in the regulation of the mitochondrial-dependent apoptosis pathway.

There is substantial experimental evidence indicating the involvement of mitochondrial dysfunction in the development of SIC. However, data concerning cardiac mitochondrial dysfunction in human sepsis are limited and inferred indirectly [3]. By delving deeper into the functions and interactions of HMGA1 in mitochondrial apoptosis, we can gain a better understanding of the mechanisms of cell apoptosis regulation and provide new insights and directions for the development of relevant therapeutic methods and drugs.

## Data Availability

Data supporting the findings of this study could be obtained from the corresponding author upon reasonable request.
